# Impact of the individual components of the metabolic syndrome and their different combinations on the prevalence of atherosclerotic vascular disease in type 2 diabetes: the Diabetes in Germany (DIG) study

**DOI:** 10.1186/1475-2840-6-13

**Published:** 2007-04-26

**Authors:** Markolf Hanefeld, Carsta Koehler, Silvina Gallo, Inge Benke, Petra Ott

**Affiliations:** 1 Center for Clinical Studies-Metabolism and Endocrinology, Science and Technology Transfer, TU Dresden, Fiedlerstrasse 34, 01307 Dresden, Germany

## Abstract

**Background:**

One of the major controversies surrounding the metabolic syndrome (MetS) in type 2 diabetes is whether its single components act synergistically as risk factors for atherosclerotic vascular disease (AVD). We aimed to answer this by evaluating the relationship, and its various combinations to AVD in comparison to single traits in a population-based study with type 2 diabetes in Germany.

**Methods and results:**

4020 unselected patients with type 2 diabetes aged 35 – 80 years. MetS was: diabetes plus ≥ 2 traits of the MetS by AHA/NHBLI definition.

AVD was: history of myocardial infarction and/or coronary revascularization and/or stroke. The occurrence of AVD in relation to overall MetS/single traits/combinations was presented as OR (95% CI). Multiple logistic regression, including established cardiovascular risk factors, modeled their associations.

The prevalence of overall MetS was 74.4% and the OR for AVD was 1.41 (1.12–1.78), which however was higher for hypertension as single trait (OR 4.76). Different combinations of MetS presented a wide range of ORs (0.47 to 10.90) and strong sex differences. Some clusters of MetS including hypertension and low HDL-cholesterol presented a higher risk factor than single traits or their sum, whereas the others out of 11 possible carried no increased AVD risk. Multiple logistic regression showed independent association between AVD and overall MetS.

**Conclusion:**

The overall MetS in type 2 diabetes comprises 11 heterogenous clusters of traits. Overall MetS increases the risk of AVD in type 2 diabetes and individual traits in some clusters with hypertension and low HDL-cholesterol may act synergistically as risk factors particularly in women.

## Background

Type 2 diabetes is associated with a high prevalence of the metabolic syndrome (MetS) [1-3]. Since type 2 diabetes (T2DM) is a component of the American Heart Association/National Heart, Lung, and Blood Institute (AHA/NHBLI) definition of the syndrome, this finding might not be surprising. The association between diabetes, hypertension and gout had already been described in 1923 [4]. Already in the first prospective studies on people with T2DM and the MetS a close association with atherosclerotic vascular diseases (AVD) was observed [2,3].

It is commonly recognized that T2DM is associated with multiple AVD risk factors. However data on the epidemiology of MetS in unselected patients with T2DM are scarce. The AHA/NHBLI criteria [5] provide a simple tool for clinical practice that should help to recognize patients with the MetS. The risk of AVD in T2DM also depends on other factors such as sex, hypercholesterolemia, smoking habits and quality of diabetes control. Therefore many questions remain open about the epidemiology and relevance of the MetS in T2DM in different countries. Recently Kahn et al [6], in a critical appraisal, have emphasized that the MetS in T2DM has not been shown so far to have more predictive power for AVD than its individual components. With two additional traits to meet the definition of the MetS, 6 triads of combinations of traits are possible. All together we can find 11 likely combinations that meet the diagnosis of MetS in patients with T2DM and they may carry different risk for AVD. The epidemiology of these different combinations and their relationship with AVD in a population with T2DM has not been evaluated so far.

The Diabetes in Germany (DIG) study is a population-based, prospective, observational study in patients with T2DM. This report will analyse the following questions in a large cross-sectional sample:

1) What is the prevalence of the MetS by AHA/NHBLI criteria, its single traits and their combinations in a representative German population with T2DM?

2) Is the MetS an independent risk factor for AVD compared to its single components in men and women?

3) What is the AVD risk for different clusters of the MetS?

## Research design and methods

Two hundred and thirty eight practices (79 in big cities, 110 in small towns and 49 in rural areas), that represent a cross section of daily practice diabetes care in Germany, took part in the study. With respect to the distribution of sites in the federal states of Germany was found out an average frequency of 1.7 to 3.5 per 10,000 inhabitants, with exception of Saxony where the ratio was 16.9 to 10,000 resp. Inclusion criteria were clinical T2DM and age between 35 and 80 years. Exclusion criteria were: major cardiovascular event <3 months before entry (myocardial infarction (MI), stroke, amputation), heart failure NYHAIV, macro-proteinuria >300 mg/24 h or creatinine >2 mg/dl, cancer disease <5 years before entry. The participating physicians (General Practitioners and Diabetologists) were asked to include ≥ 10 consecutive patients with T2DM per site. A total of 4,331 patients were recruited of whom 311 had to be excluded due to protocol violations; thus 4,020 were included in the final analysis. Medical history and drug intake were registered by a standardized questionnaire. Body mass index (BMI) and blood pressure were measured according to a standard protocol and all laboratory measurements were done by local laboratories that held a quality control certification. The following parameters were registered as baseline characteristics: sex, age, duration of diabetes, current smoking, BMI, waist circumference, systolic and diastolic blood pressure, fasting and postprandial blood glucose, HbA_1C_, total cholesterol, LDL-cholesterol, HDL-cholesterol, triglycerides, and creatinine. The traits of the MetS were defined based on the AHA/NHBLI criteria with one modification. Obesity was defined as BMI ≥ 30 kg/m^2 ^since waist circumference was not measured routinely. Hypertension was diagnosed if blood pressure at the time of inclusion was ≥130/85 mmHg and/or the patients were treated with antihypertensive drugs. The same applies for dyslipidemia with respect to fibrates and nicotinic acid. Statines, however were not considered as equivalent of dyslipidemia.

We diagnosed triads (6) of the MetS (diabetes plus two other traits), quartets (4) (diabetes plus 3 other traits) and a quintet presenting all the 5 traits. Overall MetS was diagnosed when T2DM coexisted with ≥ 2 other traits of the MetS. Cardiovascular events were identified based on the medical history obtained by the physicians at the participating sites. The diagnosis of AVD was established if the following aggregated events were present: MI and/or coronary revascularization and/or stroke. Since the database did not allow differentiating amputations by cause we did not include peripheral vascular disease in the calculations for AVD. The Saxon Ethics Committee approved the study and informed consent was obtained from all patients.

## Statistics

All data were analyzed using statistical software SPSS Version 11.5. Baseline characteristics were presented as means (SD) for continuous variables and frequency for all categorical variables. The statistical significance of the differences by sex was assessed by t-tests or χ^2 ^tests. The probability in the occurrence of AVD in relation to the MetS, its single traits and their combinations were estimated as odds ratio (OR) (95% CI). Binary logistic regression was used to model the associations of the overall MetS, its single traits, their combinations and of major established risk factors with AVD and the test of interaction by collinearity diagnostics.

## Results

Baseline characteristics of the patients by sex are given in table 1. The study subjects had a mean (SD) age of 61.8 (8.1) years and duration of the disease of 8.4 (6.8) years. Women were significantly older. The percentage of smokers was low (15%) with significantly more smokers among men. The average BMI was significantly higher in women. Mean systolic blood pressure was 139 (17) mm/Hg, diastolic blood pressure was in the normal range (82 (9) mmHg) with no significant sex difference. The quality of diabetes control was rather good with an average HbA_1C _of 7 (1.2)% for both sexes. Males had significantly higher postprandial glucose levels than females. Average LDL-cholesterol was 3.2 (1.0) mmol/l with significantly higher levels in women. Triglycerides (2.2 (1.7) mmol/l) were significantly higher in men and the same applied to creatinine. HDL-cholesterol was significantly higher in women with an overall level of 1.3 (0.4) mmol/l.

**Table 1:** 

**Parameter**	**Total population**	**Males**	**Females**
Number	4020	2140	1880
Age (years) ^a^	61.8 (8.1)	61.2 (8.1)	62.2 (8.0)
Diabetes duration (years)	8.4 (6.8)	8.2 (6.9)	8.6 (6.7)
Current smoking (%) ^c^	15.0	18.9	10.5
BMI (kg/m^2^) ^a^	30.7 (5.2)	30.0 (4.5)	31.6 (5.8)
Blood pressure (mmHg) systolic	139 (17)	139 (17)	140 (18)
- diastolic	82 (9)	82 (9)	82 (9)
Blood glucose (mmol/l) fasting	7.6 (2.5)	7.6 (2.5)	7.5 (2.4)
- postprandial^b^	9.2 (2.8)	9.3 (2.8)	9.0 (2.7)
HbA1c (%)	7.0 (1.2)	7.0 (1.3)	7.0 (1.2)
Total cholesterol (mmol/l) ^a^	5.5 (1.2)	5.4 (1.2)	5.7 (1.2)
LDL-cholesterol (mmol/l) ^a^	3.2 (1.0)	3.1 (1.0)	3.4 (1.0)
HDL-cholesterol (mmol/l) ^a^	1.3 (0.4)	1.2 (0.3)	1.4 (0.4)
Triglycerides (mmol/l) ^b^	2.2 (1.7)	2.3 (1.8)	2.2 (1.7)
Creatinine (μmol/l) ^a^	82.9 (19.5)	89.2 (18.9)	75.7 (17.6)
Hypertension (%)	91.3	91.3	91.4
Low HDL-cholesterol (%)	9.3	10.0	8.4
Hypertriglyceridaemia (%)	55.4	56.5	54.1
Obesity (%) ^a^	49.8	44.4	55.9
Only diabetes (%)	2.4	2.6	2.2
Antihypertensive therapy (%)	72.9	71.2	74.8
Medical treatment of diabetes (%)			
- oral drugs	42.3	44.1	40.4
- insulin treatment	44.8	41.0	44.9
Therapy with statins (%) ^a^	27.9	29.3	26.2
Therapy with fibrates (%)	4.2	4.6	3.8

Baseline characteristics of the DIG population divided by sex. Data are mean (SD) and %. Gender differences ^a ^p < 0.05; ^b ^p < 0.01; ^c ^p < 0.001.

With respect to the prevalence of major cardiovascular diseases, 7.6% of the patients reported a previous MI (10.8% males and 3.8% females) and 4.3% a stroke (4.5% males and 4.1% females). The frequency of coronary revascularization was high (6.7% total, 9.8% males and 3.1% females) in relation to the frequency of MI. Coronary revascularization and MI were 2–3 times more frequent in males, but there was no significant sex difference for stroke. According to our definition 15.2% of our patients had AVD. Males had a significantly higher frequency than women: 20.0% and 9.8% resp. (p < 0.001).

Elevated blood pressure was by far the most frequent single trait of the MetS found in 91.3%. Approximately every second patient had a BMI of ≥30 kg/m^2^and more than half (55.4%) had hypertriglyceridemia. In contrast, the percentage of people with low HDL-cholesterol was less than 10%. The only significant sex difference for single traits found was in the percentage of obesity with 44.4% in men and 55.9% in women (p < 0.05). Only 2.4% of the patients had no additional trait of the MetS with no sex difference. Duration of diabetes had no significant effect on prevalence of MetS.

The prevalence of different combinations which meet the diagnosis MetS is presented in table 2. We found in 35.3% (36.4% for M, 34.1% for F) a triad representing the MetS. As expected from the high prevalence of hypertension, obesity and hypertriglyceridemia the triads with these three components were found in about any second patient in both sexes (table 2). The frequency of the other triad clusters was below 10%. The same applies for quartets. Only the quartet including hypertension, hypertriglyceridemia and obesity reached a prevalence of 31.9% with significantly higher prevalence in women (34.6%) than in men (29.5%). The frequency of the other 3 quartets was far below 10%. Only 4.4% of the patients revealed the full set of traits. Overall MetS was diagnosed in 74.4% with no significant sex difference. A comparison of the prevalence of MetS by IDF and AHA/NHBLI definition in 2993 subjects with waist measurement besides BMI revealed only minor differences for both definitions: 82.6% were diagnosed by IDF criteria without a significant sex difference.

**Table 2:** 

**Phenotype**	**Total population**	**Males**	**Females**
DM+HBP+LHDL (%)	9.7	10.6	8.6
DM+HBP+HTG (%)	55.9	57.1	54.6
DM+HBP+Obes (%)^a^	50.7	45.3	55.6
DM+LHDL+HTG (%)	8.4	8.7	7.9
DM+LHDL+Obes (%)	5.6	5.6	5.6
DM+HTG+Obes (%)^a^	33.7	31.1	36.6
DM+HBP+LHDL+HTG (%)	7.8	8.3	7.2
DM+HBP+LHDL+Obes (%)	5.3	5.5	5.2
DM+HBP+HTG+Obes (%)^a^	31.9	29.5	34.6
DM+LHDL+HTG+Obes (%)	4.7	4.8	4.6
DM+HBP+LHDL+HTG+Obes (%)	4.4	4.6	4.2
DM + one trait (%)	20.5	21.5	19.3
DM + two traits (%)	35.3	36.4	34.1
DM + three traits (%)^a^	27.2	25.1	29.5
Overall MetS (%)	74.4	73.2	75.8

Prevalence of the different phenotypes of MetS in the DIG population by sex.

Sex differences ^a ^p < 0.001.

**Figure 1:**
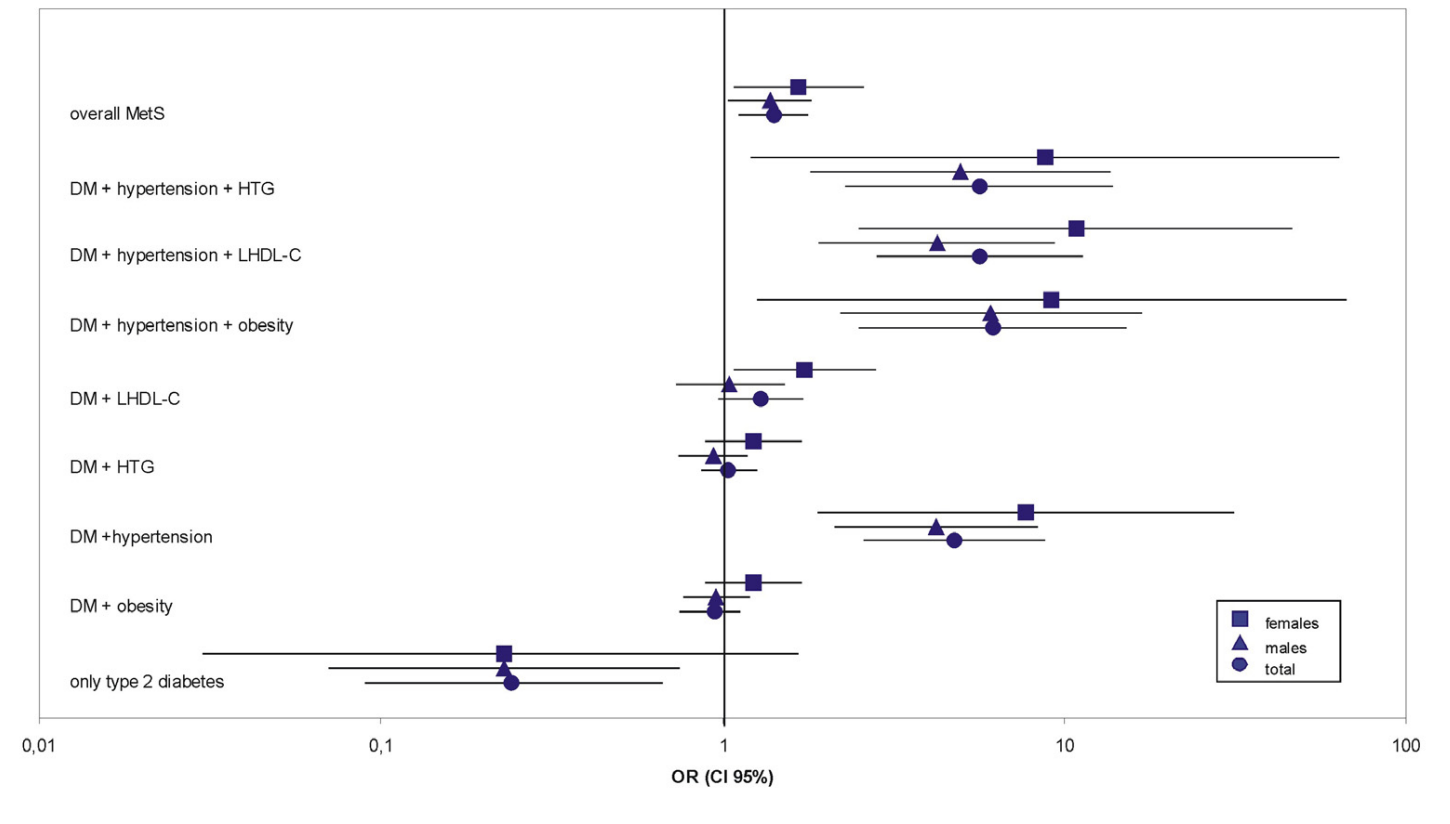
OR (95% CI) for AVD in the DIG population divided by sex. Data are presented for overall MetS, single traits and their combinations of the MetS. Only those combinations with associated AVD events are displayed. The scale of OR was log-transformed.

The ORs for AVD risk are presented in table 3. Patients without another trait had a low OR of 0.24 only, what was significant for males. Elevated blood pressure as companion increased the OR significantly to 4.22 in males and 7.69 in females. The only other single trait of significance was low HDL-cholesterol in females (OR 1.74). For triads meeting the MetS diagnosis it were the clusters with hypertension representing with the highest risk: OR 4.25 for men in combination with low HDL and 10.90 for women resp. The OR for the combination with hypertension plus hypertriglyceridemia and hypertension plus obesity was in the same range, whereas the triads with two lipids and obesity except for hypertriglyceridemia plus low HDL in females did not increase the risk for AVD. Among the quartets only the combinations including hypertension plus low HDL-cholesterol in females were associated with an increased risk (OR 1.90 and 1.92 resp.). The OR for the quintet, present in 4.4% of the patients again was only significantly increased in women (OR 2.03). Eventually, the overall MetS representing a heterogenous mix of 11 clusters meeting the diagnosis exhibited a significantly increased OR of 1.38 in men and 1.67 in women resp. As illustrated in figure 1 hypertension and less pronounced low HDL-cholesterol are the significant contributors to AVD risk whether as single additive traits or as components of the metabolic syndrome. There is no stepwise increase from triads to quintet. Women exhibit a distinctly higher risk if suffering from the MetS in any combination.

**Table 3:** 

**Phenotype**	**Total population**	**Males**	**Females**
DM alone	0.24 (0.09–0.67)	0.23 (0.07–0.75)	0.23 (0.03–1.66)
**DM+HBP**	**4.76 (2.59–8.76)**	**4.22 (2.13–8.36)**	**7.69 (1.89–31.36)**
**DM+LHDL**	1.29 (0.97–1.72)	1.05 (0.73–1.52)	**1.74 (1.08–2.81)**
DM+HTG	1.04 (0.87–1.26)	0.94 (0.74–1.18)	1.23 (0.89–1.70)
DM+Obes	0.95 (0.79–1.13)	0.96 (0.77–1.20)	1.23 (0.89–1.71)
**DM+HBP+LHDL**	**5.67 (2.84–11.31)**	**4.25 (1.92–9.41)**	**10.90 (2.51–47.46)**
**DM+HBP+HTG**	**5.64 (2.29–13.87)**	**4.96 (1.80–13.71)**	**8.78 (1.21–63.91)**
**DM+HBP+Obes**	**6.17 (2.51–15.16)**	**6.11 (2.22–16.85)**	**9.19 (1.26–66.82)**
**DM+LHDL+HTG**	1.14 (0.82–1.58)	0.85 (0.55–1.31)	**1.78 (1.07–2.96)**
DM+LHDL+Obes	0.90 (0.59–1.37)	0.54 (0.29–1.01)	1.76 (0.97–3.19)
DM+HTG+Obes	0.96 (0.79–1.17)	0.91 (0.71–1.17)	1.20 (0.86–1.68)
**DM+HBP+LHDL+HTG**	1.21 (0.87–1.69)	0.90 (0.58–1.39)	**1.90 (1.13–3.20)**
**DM+HBP+LHDL+Obes**	0.95 (0.62–1.46)	0.56 (0.30–1.04)	**1.92 (1.05–3.50)**
DM+HBP+HTG+Obes	1.01 (0.82–1.23)	0.93 (0.72–1.21)	1.29 (0.92–1.79)
DM+LHDL+HTG+Obes	0.86 (0.54–1.38)	0.47 (0.23–0.95)	1.85 (0.97–3.52)
**DM+HBP+LHDL+HTG+Obes**	0.92 (0.57–1.47)	0.49 (0.24–1.00)	**2.03 (1.06–3.87)**
**Overall MetS**	**1.41 (1.12–1.78)**	**1.38 (1.04–1.82)**	**1.67 (1.08–2.59)**

Odds ratios (95% CI) for AVD of different phenotypes of MetS in the DIG population by sex

We conducted logistic stepwise regression analysis to determine the relevance of the MetS as an independent risk factor for AVD in T2DM in the context of other major cardiovascular risk factors. We used 2 models; in the first we included the well-established risk factors: age, sex, smoking habit and LDL-cholesterol levels along with the single traits of the MetS. In the second model we considered the MetS as a whole, independently of the combination used, along with the above-mentioned established risk factors. In both models age, male sex, LDL-cholesterol levels and smoking were independently associated to AVD. In the first model T2DM with hypertension remained also independently associated to AVD as a trait of the MetS, while in the second model the MetS as a whole showed an independent association with AVD. There was no significant influence of interaction between the single traits of MetS on AVD. The calculated models predicted 85.1% in model 1 and 85.0% in model 2 resp of AVD or non AVD correctly.

## Discussion

We found in this large population based study of patients with T2DM in Germany a prevalence of the overall MetS according to AHA/NHBLI criteria of 74.4% with no sex difference. However, the analysis of the 11 possible combinations which meet the diagnosis MetS reveals a striking heterogeneity in the prevalence of phenotypes and their ORs as AVD risk factors resp. Furthermore, we observed a distinct sex difference with respect to ORs for single traits as well as for all phenotypes of the MetS.

The prevalence of overall MetS in DIG is comparable to other studies with T2DM in Europe, also they use different definitions [2,7-11] and slightly lower than the prevalence reported from NHANES_III _[1]. Only 2.4% of the patients had no other trait, what fits well to the other publications and illustrates the close overlap of T2DM with the components of the MetS [1].

As already described in other epidemiological investigations with T2DM and MetS hypertension was the most frequent co-morbidity with not less than 91.3%, with no sex difference. In a recently published study triglycerides were the strongest predictor to indicate the presence of MetS among patients with type 2 diabetes of a outpatient clinic [12]. In contrast to the high prevalence of hypertriglyceridemia (55.4%) only 9.3% had decreased HDL-cholesterol levels. The rather high level of HDL-cholesterol may be explained by the fact that the patients of the DIG study reached at average HbA_1_C level of 7% and that 44.8% of them were receiving insulin treatment, which is known to increase HDL-cholesterol [13]. Obesity was significantly more present in women (55.9%) than men (44.4%). When we compared the prevalence of central obesity diagnosed by waist circumference with obesity by BMI we did not find a large difference in the prevalence. Thus our data should not be biased by missing waist circumference.

For the first time we present data on the distribution and prevalence of the 11 possible clusters hidden in the unspecified diagnosis overall MetS. In the DIG sample 62.5% of the people had two or three additional traits (triads, quartets) and only 4.4% had a complete set of components of MetS. As expected from the high prevalence of hypertension, hypertriglyceridemia and obesity the prevalence of triads with these components was high: 55.9% for combination with hypertension plus hypertriglyceridemia, 50.7% for combination with hypertension plus obesity and 33.7% for hypertriglyceridemia and obesity as companions. Among quartets only the combination with these three traits was of relevance (31.9%). Of notice was the low frequency of remaining phenotypes with prevalences between 4.7 and 9.7%.

Our data on OR for AVD suggest on the first look that the overall MetS is associated with an increased risk: OR for men 1.38 and for women 1.67 resp., but the corresponding ORs for hypertension as single trait were 4.22 and 7.69 resp. In the Botnia study the MetS by WHO definition in patients with T2DM did not increase the AVD mortality (RR 1.15 CI 0.68–1.94), while some of its individual components such as hypertension and albuminuria were highly significant predictors of cardiovascular events. Therefore a critical appraisal put a question to the clinical relevance of MetS [6]. Based on ADA/NHBLI criteria in T2DM the diagnosis of MetS can be made for 6 triads, 4 quarters and one quintet of components of the MetS. As shown in tab. 3 and figure 1 the ORs of these 11 phenotypes vary in a wide range from 0.47 to 10.90 depending on individual components and sex. Thus the clusters of the MetS including hypertension and low HDL-cholesterol are clearly associated with a higher AVD risk than any single trait or their sum showing OR for these triads of 5.67, 5.64 and 6.17 resp. On the other hand some clusters in the DIG study revealed ORs below average risk. This was the case for specified phenotypes if they did not include hypertension and low HDL-cholesterol as components. In a prospective investigation in patients with coronary angiography [14] dyslipidemia and hypertension were found to be the hard core of MetS. Our investigation demonstrates that the AVD risk strongly depends on the individual components which are the basis of MetS diagnosis. This varies between study populations depending on age, sex, race and other factors and for people with and without diabetes, what may explain discrepancies between epidemiological studies with MetS as AVD risk factor. As shown in the DIG study women with single traits and/or any cluster of the MetS carry at average a twice higher AVD risk than men.

In the DIG study overall MetS could be confirmed as independent AVD risk factor in multiple regression analysis including established risk factors such as LDL-cholesterol, age, sex and smoking. The predictive power of this model was as good as for multivariate analysis taking the single traits into the model. We did not find a statistical interdependence of the single traits as risk factors. In a prospective, community-based study, using modified AHA/NHBLI definition, in patients with T2DM the increase in the number of traits was associated with higher CVD rates [15]. Another prospective study in women >65 years with diabetes concluded that the association between the MetS and mortality (mainly AVD) was stronger for the overall syndrome than for each individual component [16].

There are some limitations to our analysis. We made a cross-sectional evaluation and our results are subject, therefore, to survival bias. Some of the combinations of traits were present in a very low number so that the ORs could not be calculated or resulted no significant. In addition, the diagnosis of AVD was based on medical history and information obtained by home physicians without the participation of an adjudicator committee. On the other hand, this population sample represents the real-world scenario of T2DM in Germany.

The metabolic syndrome is not more or less than a concept for an integrated approach to a cluster of metabolic diseases with hypertension [17]. This study was not designed to prove the concept. For this prospective studies are urgently needed.

In conclusion the MetS was found to be an independent risk factor for AVD in the DIG study together with established risk factors such as age, sex, LDL-cholesterol and smoking. The prognostic power was equal to models with the single trait as categorical variable. Hypertension as single variable had a higher AVD risk than overall MetS. However, triads of the MetS including hypertension and/or low HDL-cholesterol had twice as high a risk as hypertension alone. There are however other phenotypes of the MetS with no increased risk. Thus the metabolic syndrome comprises heterogenous clusters with respect to AVD risk. The data of the DIG study support the concept to focus on multiple risk factor strategy to prevent AVD in diabetes.

## Competing interests

The author(s) declare that they have no competing interests.
